# Supplementary material for: Association between oral microbiome and seven types of cancers in East Asian population: a two-sample Mendelian randomization analysis
Source: Front Mol Biosci. 2023 Nov 21;10:1327893. doi: 10.3389/fmolb.2023.1327893 (PMC10702768; doi:10.3389/fmolb.2023.1327893)
Supplement: Supplementary file 1 [file DataSheet1.ZIP › Supplementary Materials/MR plots for tongue/tongue═╝/Ovarian cancer/pheno.3_to_ovarian cancer_leave_one_out.pdf]

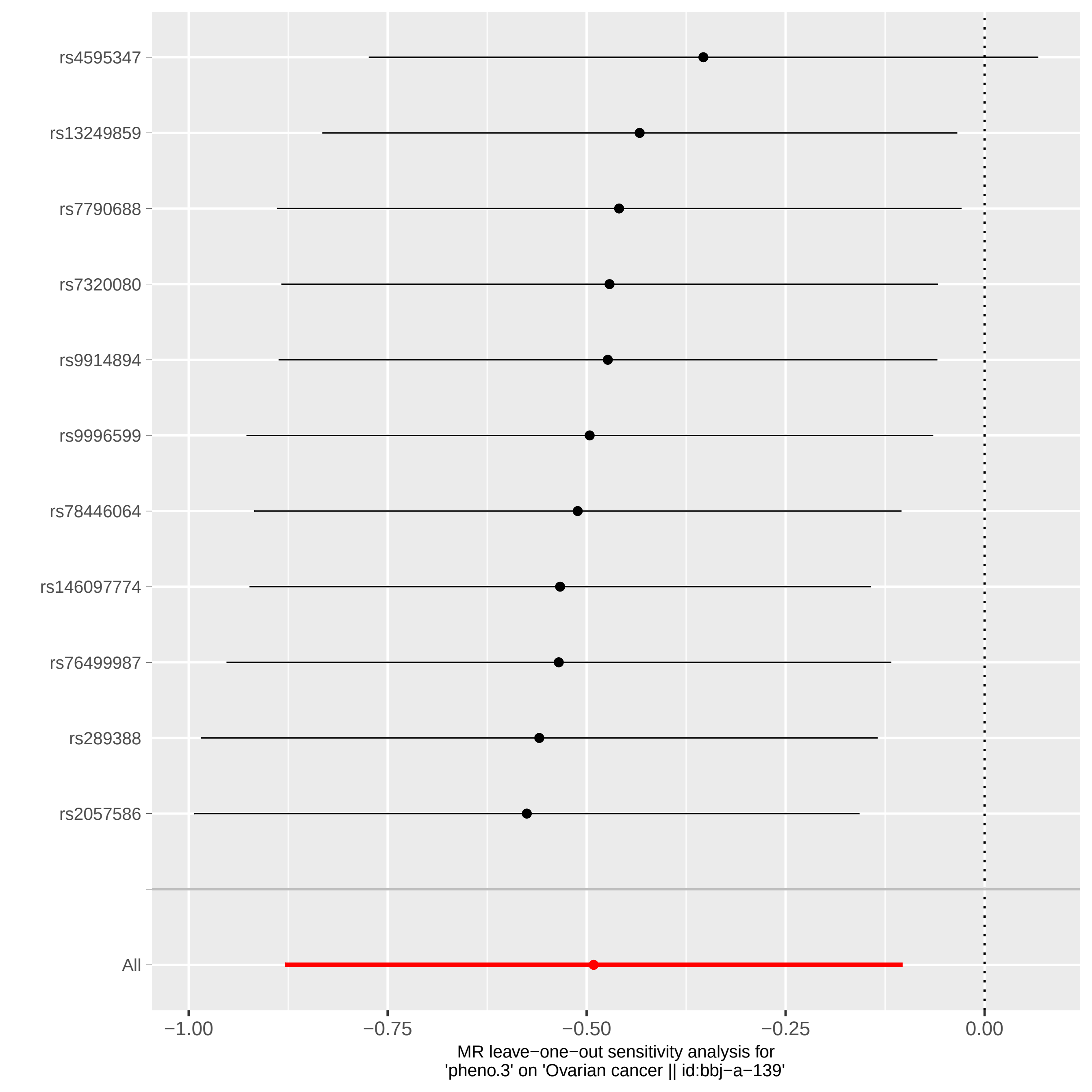

rs4595347
rs13249859
rs7790688
rs7320080
rs9914894
rs9996599
rs78446064
rs146097774
rs76499987
rs289388
rs2057586
All
−1.00
−0.75
−0.50
−0.25
0.00
MR leave−one−out sensitivity analysis for
'pheno.3' on 'Ovarian cancer || id:bbj−a−139'
